# Combination Adjuvants Enhance Recombinant Protein Vaccine Protection against Fungal Infection

**DOI:** 10.1128/mBio.02018-21

**Published:** 2021-08-17

**Authors:** Marcel Wüthrich, Hannah E. Dobson, Cleison Ledesma Taira, Uju Joy Okaa, Lucas dos Santos Dias, Marcos Isidoro-Ayza, Nikolai Petrovsky, Bruce S. Klein

**Affiliations:** a Department of Pediatrics, University of Wisconsin School of Medicine and Public Health, University of Wisconsin—Madison, Madison, Wisconsin, USA; b Internal Medicine, University of Wisconsin School of Medicine and Public Health, University of Wisconsin—Madison, Madison, Wisconsin, USA; c Medical Microbiology and Immunology, University of Wisconsin School of Medicine and Public Health, University of Wisconsin—Madison, Madison, Wisconsin, USA; d Vaxine, Pty. Ltd., Warradale, Australia; e College of Medicine and Public Health, Flinders University, Bedford Park, Australia; Albert Einstein College of Medicine

**Keywords:** fungi, T cells, adjuvants, immunization

## Abstract

The development of effective vaccines against fungal infections requires the induction of protective, pathogen-specific cell-mediated immune responses. Here, we asked whether combination adjuvants based on delta inulin (Advax) formulated with Toll-like receptor (TLR) agonists could improve vaccine protection mediated by a fungal recombinant protein, Bl-Eng2 (i.e., *Blastomyces* endoglucanase 2), which itself harbors an immunodominant antigen and dectin-2 agonist/adjuvant. We found that Bl-Eng2 formulated with Advax3 containing TLR9 agonist or Advax8 containing TLR4 agonist provided the best protection against pulmonary infection with Blastomyces dermatitidis, being more effective than complete Freund’s adjuvant or Adjuplex. Advax3 was most efficient in inducing gamma interferon (IFN-γ)- and interleukin-17 (IL-17)-producing antigen-specific T cells that migrated to the lung upon Blastomyces dermatitidis infection. Mechanistic studies revealed Bl-Eng2/Advax3 protection was tempered by neutralization of IL-17 and particularly IFN-γ. Likewise, greater numbers of lung-resident T cells producing IFN-γ, IL-17, or both IFN-γ and IL-17 correlated with fewer fungi recovered from lung. Protection was maintained after depletion of CD4^+^ T cells, partially reduced by depletion of CD8^+^ T cells, and completely eliminated after depletion of both CD4^+^ and CD8^+^ T cells. We conclude that Bl-Eng2 formulated with Advax3 is promising for eliciting vaccine-induced antifungal immunity, through a previously uncharacterized mechanism involving CD8^+^ and also CD4^+^ T cells producing IFN-γ and/or IL-17. Although no licensed vaccine exists as yet against any fungal disease, these findings indicate the importance of adjuvant selection for the development of effective fungal vaccines.

## INTRODUCTION

Despite medical advances, invasive fungal infections have skyrocketed over the last decade. They pose a mounting health threat in immunocompetent and -deficient hosts, with worldwide mortality rates ranking 7th, even ahead of tuberculosis (1, 2). The development of safe and effective vaccines remains a major hurdle for fungal prophylaxis. Currently, there are no licensed vaccines against fungi. Subunit vaccines are ideal candidates since they are safe for use in immunocompromised individuals in whom live attenuated vaccines are contraindicated. However, subunit vaccines generally require formulation with a potent adjuvant to be effective. The lack of appropriate adjuvants is one major impediment to developing safe and effective vaccines against fungal pathogens, as is a lack of understanding of the best type of immune response a fungal vaccine would need to elicit to induce maximal protection.

Adjuvants can provide multiple functions, including delivery of antigens (Ags) to antigen-presenting cells (APCs), formation of antigen depots, chemotaxis, enhanced dendritic cell (DC) function, and B- or T-cell costimulation. Adjuvants can be combined for enhanced action, such as the combination of innate immune activators, such as Toll-like receptor (TLR) ligands, with more traditional adjuvant components, such as aluminum hydroxide, that act as antigen carriers.

Adaptive immunity mediated by CD4^+^ T cells plays the major role in resolution of fungal infections (3, 4), as evidenced by the high incidence of invasive fungal infections in patients with impaired CD4^+^ T-cell immunity. CD4^+^ T cells confer resistance by secretion of T-helper 1 (Th1) and Th17 cytokines, such as gamma interferon (IFN-γ), tumor necrosis factor alpha (TNF-α), granulocyte-macrophage colony-stimulating factor (GM-CSF), and interleukin-17A (IL-17A), which activate neutrophils, monocytes, macrophages, and DCs for fungal clearance (4–9). Thus, in order to be protective, adjuvants used for fungal vaccination may need to generate a strong Th1 and Th17 cell immune response.

Advax is based on plant-derived inulin, a polysaccharide that when formulated into delta inulin particles and coadministered with subunit antigens (Ags) induces Ag-specific Th1, Th2, and Th17 T-cell responses (10–12). Among its immunological effects, Advax induces a strong chemotactic effect, resulting in the recruitment of leukocytes to the site of vaccination, and engenders vaccine-induced protection against viral and bacterial pathogens (10–18). Advax has not been previously investigated in fungal vaccines. Formulation of Advax with TLR agonists enables additional modulation of T-cell phenotypes, which may further enhance protective immunity. For example, formulation of the recombinant fusion protein CysVac containing Ag85B and CysD with Advax and the TLR9 agonist CpG drove an Ag-specific Th1 and Th17 cell response and provided superior protection against aerosol Mycobacterium tuberculosis infection (13).

Here, we sought to formulate a recombinant protective fungal antigen, *Blastomyces* endoglucanase 2 (Bl-Eng2), with Advax and various TLR agonists to determine the T-helper phenotypes of Ag-specific T cells that best provide protection from fungal infection. We found that Advax3, which contains the TLR9 agonist CpG, drove a strong, balanced Th1 and Th17 cell response and yielded optimal protection against pulmonary infection with the fungus Blastomyces dermatitidis. Advax3 vaccine-induced resistance was mediated by IFN-γ- and IL-17-producing CD4^+^ and CD8^+^ T cells.

## RESULTS

### Adjuvant formulations influence antifungal T-cell development and resistance to fungal infection.

To assess the extent to which distinct adjuvant formulations might influence vaccine-induced protection against fungi, we tested nine different adjuvant formulations (Advax1 to -9) over multiple experiments (Table 1). We compared these Advax formulations to CpG, complete Freund’s adjuvant (CFA), and Adjuplex adjuvant. We formulated the adjuvants with the protective CD4 T-cell antigen Bl-Eng2 (19), vaccinated mice twice by the subcutaneous (s.c.), route and challenged them with a pulmonary infection of B. dermatitidis 2 weeks after the boost.

**TABLE 1:** Overview of vaccine adjuvant formulations

Adjuvant	Formulation
Advax1	Inulin polysaccharide adjuvant alone with no TLR agonist
Advax2	Advax1 + 10 μg TLR9 agonist (CpG55.2)
Advax3	Aldeltin (special formulation containing alum OH) + 10 μg TLR9 agonist (CpG55.2)
Advax4	Advax1 + 10 ng TLR7/8 agonist
Advax5	Advax1 + 200 ng TLR4 agonist
Advax6	Advax1 + 5 μg TLR3 agonist
Advax7	Advax1 + 5 μg TLR2 agonist
Advax8	Advax1 + 1 μg TLR4 agonist (5 times more TLR4 agonist than Advax5)
Advax9	Inulin polysaccharide alone with no TLR agonist-improved antigen binding capacity vs Advax1

**FIG 1:**
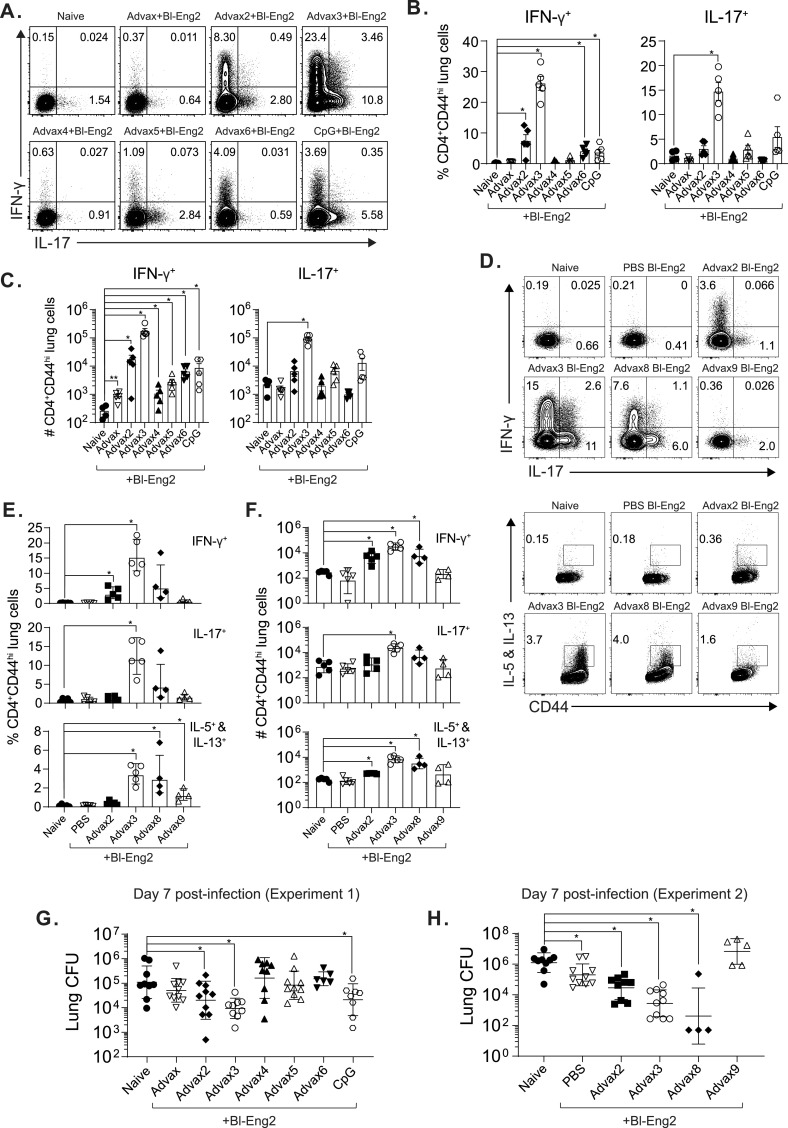
Adjuvant formulations elicit variable Ag-specific T-cell responses and protection against fungal infection. C57BL6 mice were vaccinated s.c. with Advax formulations or CpG and 10 μg of soluble Bl-Eng2 protein twice, 2 weeks apart. Two weeks after the boost, animals were challenged with 2 × 10^4^ CFU of B. dermatitidis yeast. Seven days postchallenge, mouse lungs were harvested. Dot plots display IFN-γ and IL-17 cytokine production after 5 h of stimulation with 5 μM Bl-Eng2 peptide and anti-CD28. (A to C) Corresponding dot plots and bar graphs show the frequency (A and B) and absolute numbers (C) of cytokine-producing CD44^hi^ CD4^+^ T cells (*n* = 5 mice/group). (D to F) Repeat experiment with the addition of Advax8 and -9. *, *P* < 0.05. (G and H) Resistance to infection as determined by lung CFU. The graph shows the geometric mean with standard deviation (*n* = 8 to 10 mice/group).

At day 7 postinfection, which coincides with the peak influx of primed T cells (20) into the lung, we enumerated the frequencies and numbers of lung cytokine-producing T cells following *ex vivo* stimulation with Bl-Eng2 peptide (19). The tested adjuvant formulations had various effects on the frequencies and cytokine profiles of the responding T cells. In the first experiment, the frequencies and numbers of lung IFN-γ- and IL-17-producing CD4^+^ T cells postchallenge were significantly increased by the addition of Advax2, -3, and -6 and CpG compared to naive animals (Fig. 1A to C). While Advax2 and -6 and CpG induced predominantly single-positive IFN-γ- or IL-17-producing CD4^+^ T cells, with only a small population of double-positive IFN-γ and IL-17 CD4^+^ T cells (0.49, 0.03, and 0.35%, respectively), Advax3 was the only adjuvant that induced a relatively large population of double-positive IFN-γ- and IL-17-producing cells (3.46%).

We performed a second experiment to reproduce the initial results with Advax2 and -3 and also investigate two new adjuvant formulations, Advax8 and -9. Advax3 again was associated with the highest numbers of IFN-γ- and/or IL-17-secreting CD4^+^ T cells in the lung postchallenge and concomitant increase in IL-5- and IL-13-secreting T cells (Fig. 1D to F), consistent with a balanced enhancement of the Th1, Th2, and Th17 arms of the adaptive immune response. Advax2, as seen previously, was associated with a significant increase in IFN-γ-producing CD4^+^ T cells in the lung postchallenge, consistent with a Th1 bias. Advax8, like Advax3, was associated with increases in all CD4^+^ T-cell populations, although the increase in IL-17-producing CD4^+^ T cells did not reach significance. Advax9 was only associated with an increase in the frequency of IL-5- and IL-13-producing CD4^+^ T cells, consistent with an overall Th2 bias. Advax4, -5, -6, and -7, which contained delta inulin plus TLR-7/8, TLR-4, TLR-3, and TLR-2 agonists, respectively, had modest effects if any and were not further pursued.

In the first experiment, formulation of Bl-Eng2 with Advax2 or -3 and CpG significantly reduced lung CFU compared to unvaccinated control mice, with the greatest CFU reduction observed in the Advax3 group (Fig. 1G). The level of vaccine protection correlated with the frequencies of single- and double-positive IFN-γ- and IL-17-producing CD4 T cells and was highest for Advax3, followed by Advax2 and then CpG (Fig. 1C and F and see Fig. 3A below). Notably, those groups that had no increase in double-positive IFN-γ- and IL-17-producing CD4^+^ T cells (Advax4, -5, and -6) also had no significant reduction in lung CFU (Fig. 1G).

In the second experiment, formulation of Bl-Eng2 with Advax2, -3, and -8 significantly reduced lung CFU compared to unvaccinated control mice, with the greatest overall CFU reduction seen with Advax3 and -8 (Fig. 1H). Interestingly, vaccine formulated with Advax9 showed a polarized Th2 response and no protection, suggesting that a Th2-biased immune response is not beneficial in protecting against lung fungal infection. In summary, Advax3 elicited the highest number of double IFN-γ- and IL-17-producing CD4^+^ T cells and reduced lung CFU efficiently compared to the other Advax and CpG formulations.

### Advax3 compared to Adjuplex and Freund’s adjuvant.

**FIG 2:**
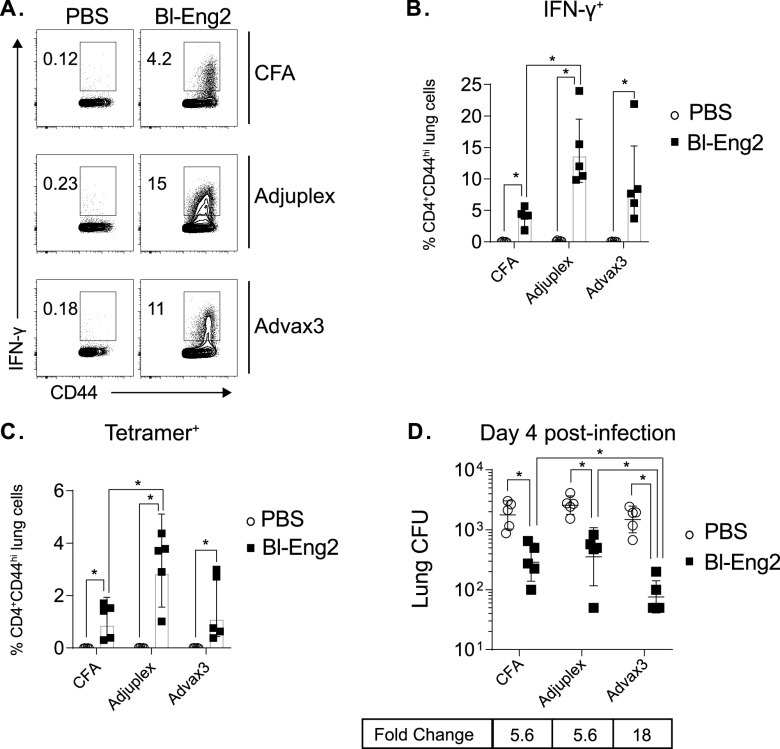
Advax3 provides enhanced fungal protection compared to Adjuplex and Freund’s adjuvant. C57BL6 mice were vaccinated s.c. with 10 μg of soluble Bl-Eng2 protein in either Freund’s adjuvant (CFA/IFA), 10% Adjuplex, or Advax3 three times, spaced 2 weeks apart. Two weeks after the last boost, the mice were challenged with 2 × 10^4^ CFU of B. dermatitidis yeast. (A and B) At day 4 postinfection, lung T cells were stimulated with Bl-Eng2 peptide, and intracellular IFN-γ was detected by flow cytometry. (C) Frequency of Bl-Eng2 peptide tetramer^+^ T cells. (D) Lung CFU. The graph shows the geometric mean with standard deviation and calculated fold change from unvaccinated controls (*n* = 5 mice/group). *, *P* < 0.05 versus unvaccinated control mice.

We next investigated how Advax3-mediated fungal protection compared to protection by other adjuvants that we, and others, have successfully used to engender subunit vaccine-induced fungal protection. We immunized mice with Bl-Eng2 formulated in Freund’s adjuvant, Adjuplex, or Advax3. All three adjuvants activated and expanded expression of IFN-γ-producing Bl-Eng2-specific CD4^+^ T cells. The greatest increase was observed in the Adjuplex group, followed by the Advax3 and CFA groups, respectively (Fig. 2A to C). This result was paralleled by the frequency of Bl-Eng2 tetramer-positive CD4^+^ T cells in the lung, which again showed the greatest increase in the Adjuplex group, followed by the Advax3 and CFA groups, respectively. However, following infection, Advax3 was associated with the greatest reduction in lung CFU (18-fold) day 4 postchallenge compared to Adjuplex and CFA adjuvanted vaccines (both 5.6-fold) Fig. 2D).

### Advax3 mediates protection by CD4^+^ and CD8^+^ T cells and IFN-γ and IL-17.

**FIG 3:**
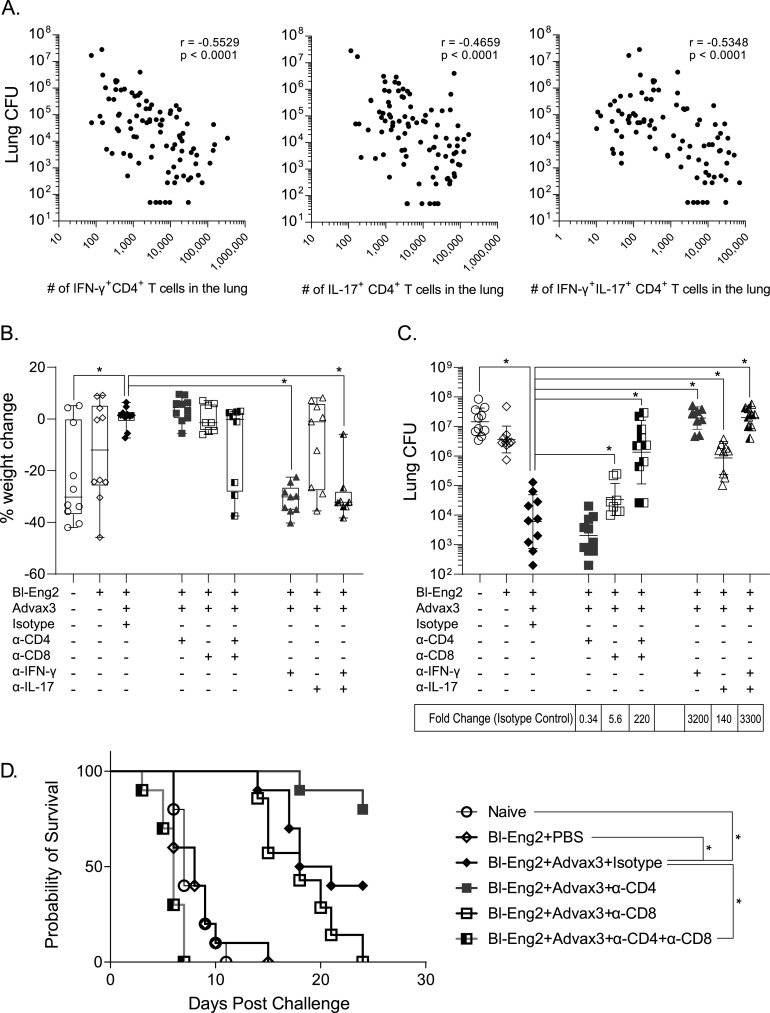
Mechanisms of Advax3-mediated protection. C57BL6 mice were vaccinated s.c. with 10 μg of soluble Bl-Eng2 protein and Advax3 two times, spaced 2 weeks apart. Two weeks after the last boost, a cohort of mice were challenged and analyzed for a correlation of cytokine-producing lung CD4^+^ T cells at day 4 postinfection (*x* axis) and lung CFU at 2 weeks postinfection (*y* axis) (A). A second cohort of mice was depleted of CD4 and/or CD8 T cells or neutralized with anti-IFN-γ or anti-IL-17 MAb and subsequently challenged with 2 × 10^4^ CFU of B. dermatitidis yeast. Two weeks postinfection, the mice were sacrificed and analyzed for weight loss (B) and lung CFU (C). The graph shows the geometric mean with standard deviation and calculated fold change from unvaccinated controls (*n* = 10 mice/group). *, *P* < 0.05 versus vaccinated control mice treated with rat IgG. A second cohort of mice (*n* = 10 mice/group) was also analyzed for survival for up to 24 days postchallenge (D). *, *P* < 0.05 versus IgG isotype control-treated animals.

We further analyzed the relationship between lung cytokine-producing CD4^+^ T cells and fungal protection. In Bl-Eng2- and Advax3-vaccinated mice, we found a significant, strong correlation between the number of single- and double-cytokine (IFN-γ and IL-17)-producing, Bl-Eng2-specific CD4^+^ T cells in the lung after infection and the reduction in lung CFU (Fig. 3A). We thus sought to investigate whether Advax3-mediated protection is dependent on Th1 and/or Th17 cells. In mice that had been vaccinated with Bl-Eng2 and Advax3, we used anticytokine antibodies to neutralize IFN-γ and/or IL-17 prior to challenge and throughout the course of infection. Separately, to further determine which T-cell population might play a key role in vaccine protection, we used antibodies to deplete CD4^+^ or CD8^+^ T cells alone or together in Bl-Eng2- and Advax3-immunized mice. Two weeks postinfection, when unvaccinated controls were moribund, all the mice were sacrificed and analyzed for weight loss and lung CFU.

Vaccinated mice that were depleted of both CD4^+^ and CD8^+^ T cells or neutralized for either IFN-γ or IL-17 or both IFN-γ and IL-17 lost significant amounts of weight and had significantly increased lung CFU compared to Bl-Eng2- and Advax3-vaccinated mice that received the isotype control antibody (Fig. 3B and C). Contrary to our expectation, depletion of CD4^+^ T cells alone had little effect on vaccine reduction of lung CFU. In contrast, depletion of CD8^+^ T cells significantly increased lung CFU by 5.6-fold, impairing but not eliminating vaccine protection. Depletion of both CD4^+^ and CD8^+^ T cells abolished protection against weight loss and lung burden of fungi (e.g., CFU), with these double-depleted mice having lung CFU values similar to those of naive mice or controls vaccinated with Bl-Eng2 alone (Fig. 3B and C). Neutralization of IFN-γ resulted in a complete loss of vaccine protection, whereas neutralization of IL-17 had a significant but lesser effect on protection.

We also investigated the impact of CD4^+^ and CD8^+^ T-cell depletion on the survival of mice that had been vaccinated with Bl-Eng2 and Advax3 (Fig. 3D). Naïve controls and animals immunized with Bl-Eng2 antigen alone succumbed to infection by day 14. In contrast, mice immunized with Bl-Eng2 plus Advax3 all survived to day 14, before some became ill, with overall 50% survival at day 24. Depletion of CD8^+^ T cells did not reduce early survival compared to rat IgG control-treated vaccinated mice, but ultimately 100% of CD8^+^ T-cell-depleted mice succumbed to infection, compared to 60% of the rat IgG control-treated vaccinated mice. Surprisingly, depletion of CD4^+^ T cells increased survival compared to that of rat IgG-treated controls, resulting in over 80% survival at 24 days. Depletion of both CD4^+^ and CD8^+^ T cells resulted in a complete loss of vaccine-induced protection, with all animals in this group succumbing to disease by day 8. Taken together, our results indicate a complex situation where not only CD8^+^ T cells, but also CD4^+^ T cells, together with IFN-γ and IL-17, all play roles in Advax3-driven vaccine protection. CD4^+^ T cells may potentially function in a double-edged manner, demonstrating the ability to both enhance and diminish vaccine protection.

To investigate whether the resistance phenotype due to CD4^+^ T-cell depletion can be explained by regulatory T-cell (Treg) elimination or altered CD8^+^ T-cell numbers and function, we analyzed the cellular and molecular perturbations of lung T cells at day 4 postinfection. Depletion CD4^+^ T cells in mice vaccinated with Advax3 plus Bl-Eng2 increased the number of total and activated (CD44^+^) CD8^+^ T cells (see Fig. S1A and B in the supplemental material), the tissue residency of activated CD8^+^ T cells (Fig. S1C and D), and the frequencies and numbers of IFN-γ- and IL-17-producing CD8^+^ T cells (Fig. S1E and F). Histological analysis of 12 random lung sections from each of the two treatment groups (rat IgG and CD4 depleted) revealed slightly, but insignificantly, less inflammation in the CD4-depleted group, with three sections showing 25 to 33% of inflamed lung versus five sections for the IgG group.

10.1128/mBio.02018-21.1 FIG S1 The impact of CD4^+^ and Treg cell depletion on CD8^+^ T cells (Fig. 3). C57BL6 mice (*n* = 5 mice/group) were vaccinated s.c. with 10 μg of soluble Bl-Eng2 protein and Advax3 two times, spaced 2 weeks apart. Two weeks after the boost, the mice were treated with anti-CD4 MAb (for CD4^+^ T-cell depletion) on the day of infection or with anti-CD25 MAb at days −7 and −4 of infection and anti-CTLA4 MAb at day 0 postinfection. At day 4 postinfection, the numbers of total (A) and activated (CD44^+^) (B) CD8^+^ T cells, tissue distribution (C and D), and frequencies (E and F) and numbers (G) of cytokine-producing CD8^+^ T cells were analyzed. *, *P* < 0.05 versus IgG isotype control-treated animals. Download FIG S1, EPS file, 2.9 MB. Copyright © 2021 Wüthrich et al. 2021 Wüthrich et al. https://creativecommons.org/licenses/by/4.0/ This content is distributed under the terms of the Creative Commons Attribution 4.0 International license.

We separately targeted Treg cells by treating Advax3- plus Bl-Eng2-vaccinated mice with anti-CD25 monoclonal antibody (MAb) at days −7 and −4 of infection and anti-CTLA4 MAb at the time of infection and weekly thereafter as described previously (21) and measured cellular responses and survival. Treg depletion reduced the frequency of Foxp3^+^ T cells in the lung at day 4 postinfection by 50% (see Fig. S2A in the supplemental material) and increased the frequency and numbers of IFN-γ-producing CD4^+^ T cells in the lung (Fig. S2B and C). However, anti-CD25/CTLA4 Ab treatment did not impact the numbers (Fig. S1A and B) or function (Fig. S1E to G) of the CD8^+^ T cells or mouse survival. (The mean durations of survival of the rat IgG control-treated versus anti-CD25/CTLA4-treated Advax3- plus Bl-Eng2-vaccinated mice were 18 ± 3 and 17 ± 3 days postinfection, respectively.) In summary, depletion of CD4^+^ T cells, but not Treg cells, increased CD8^+^ T-cell numbers and function, which could account for increased survival.

10.1128/mBio.02018-21.2 FIG S2 The impact of Treg cell depletion on CD4^+^ T cells and confirmation of CD4^+^, CD8^+^, and CD4^+^ and CD8^+^ T-cell depletion (Fig. 3). C57BL6 mice (*n* = 5 mice/group) were vaccinated s.c. with 10 μg of soluble Bl-Eng2 protein and Advax3 two times, spaced 2 weeks apart. (A to C) Two weeks after the boost, the mice were treated with anti-CD25 MAb at days −7 and −4 of infection and anti-CTLA4 MAb at day 0 postinfection. At day 4 postinfection, the frequencies of Foxp3^+^ (A) and cytokine-producing CD44^+^ CD4^+^ T cells (B) and numbers (C) of cytokine-producing CD4^+^ T cells were analyzed. *, *P* < 0.05 versus IgG isotype control-treated animals. (D and E) Verification of T-cell depletion. Two weeks after the boost, the mice were treated with anti-CD4 or anti-CD8 MAb alone and anti-CD4 and anti-CD8 MAbs together on the day of infection. At day 4 postinfection, the frequencies (D) and numbers (E) of targeted T-cell subsets were analyzed by FACS. Download FIG S2, EPS file, 2.9 MB. Copyright © 2021 Wüthrich et al. 2021 Wüthrich et al. https://creativecommons.org/licenses/by/4.0/ This content is distributed under the terms of the Creative Commons Attribution 4.0 International license.

## DISCUSSION

The hallmark of an effective adjuvant is the ability to elicit the type of adaptive immune response that mediates protective immunity to specific pathogens. In this study, we tested whether Advax inulin-based adjuvant formulations were able to enhance vaccine-induced protection against fungal infection mediated by the protective subunit antigen Bl-Eng2 (19). We found that formulating Bl-Eng2 with Advax alone did not augment vaccine protection, whereas the combination of Advax with some TLR agonists but not others did enhance vaccine protection. Combining Bl-Eng2 with Advax3 for vaccination yielded the largest recruitment of Ag-specific Th1 and Th17 cells into the lungs of vaccinated mice following fungal infection. Since vaccine resistance to fungi is principally thought to be mediated by Th1 and Th17 cells (3, 4), we accordingly found that Advax3, which recruited these cells, was most efficient in reducing lung CFU, retarding weight loss, and prolonging survival of vaccinated mice challenged with B. dermatitidis.

Both Advax2 and -3 contain the TLR9 agonist CpG, which yielded a strong induction of IFN-γ-producing CD4^+^ T cells. Advax3 induced even greater IFN-γ and IL-17 production by Bl-Eng2-specific T cells compared to Advax2, suggesting that the formulation of Advax3 containing aluminum hydroxide (Table 1) is responsible for the additive effects. In keeping with its ability to increase Th1 and Th17 cell responses, the Advax3 formulation yielded the best vaccine protection. CpG alone combined with Bl-Eng2 also yielded a mixed Th1/Th17 cell response and reduced lung CFU, although the combination of Advax with CpG (as in Advax2 and -3) was more effective in driving protective T-helper responses and vaccine protection.

Advax8 containing a TLR4 agonist also induced a mixed Th1/Th17 cell response and reduced lung CFU efficiently. The TLR4 adjuvant effect was dose dependent, as Advax5, which contained 5 times less TLR4 agonist, showed reduced production of IFN-γ and IL-17 by CD4^+^ T cells and did not reduce lung CFU compared to Advax8. Formulations containing TLR2, TLR3, and TLR7/8 agonists were likewise poorly effective at best. Taken together, our data strongly suggest that inclusion of TLR9 (CpG), or possibly TLR4 agonists, with Advax boosts the induction of Ag-specific Th1 and Th17 cells, which are tightly linked with vaccine-induced protection against fungi.

Neutralization of IFN-γ completely abolished vaccine-induced protection, whereas neutralization of IL-17 only partially attenuated this protection, indicating the relative importance of IFN-γ to Advax3-mediated vaccine protection. Depletion of both CD4^+^ and CD8^+^ T cells during the expression phase of the vaccine immune response mirrored the phenotypes of IFN-γ and IL-17 neutralization, suggesting that IFN-γ and IL-17 produced by T cells mediate Advax3 vaccine resistance. Depletion of CD8^+^ T cells increased lung CFU and reduced survival compared to isotype control antibody-treated mice, whereas depletion of CD4^+^ T cells did not reverse the vaccine effect on lung CFU and actually improved overall survival. These results indicate that CD8^+^ T-cell subsets are the primary contributor to Advax3 vaccine immunity, but with some CD4^+^ T cells still able to compensate for and provide protection in the absence of CD8^+^ T cells. Notably, subsets of CD4^+^ T cells are able to produce IFN-γ and IL-17, and it is likely these subsets that are able to promote protection in the absence of CD8^+^ T cells. Nevertheless, the data indicate that another subset of CD4^+^ T cells contributes to disease pathogenesis, given the increased survival of vaccinated mice after CD4^+^ T-cell depletion. As adjuvant groups that mounted solely Th2 cytokine responses showed minimal protection (e.g., Advax9), we speculate that CD4^+^ T cells may have a dual role in vaccine protection, with Th1 and Th17 subsets contributing to protection via IFN-γ and IL-17 production and the Th2 subset contributing to disease progression via known inhibitory effects of Th2 cytokines, such as IL-4 and IL-10, on IFN-γ and IL-17 production.

The strong contribution of CD8^+^ T cells to protection conferred by Advax3 was unexpected, as we have previously observed a primary role for CD4^+^ T cells in mediating vaccine resistance. Those results were observed when we immunized mice with a live attenuated strain of B. dermatitidis or with subunit vaccines containing the conserved antigens calnexin or Bl-Eng2 together with other adjuvants (19, 22, 23). In fact, we reported a clear hierarchy in priming of CD4^+^ T cells over CD8^+^ T cells when using the live attenuated strain to induce vaccine immunity (22, 24). In one study, we noted that depletion of CD4^+^ T cells, but not CD8^+^ T cells, during the vaccine effector phase abolished vaccine protection (22). In a second study, we reported differential requirements by CD4^+^ and CD8^+^ T cells for costimulation by the CD40/CD40L axis during the vaccine priming phase (24). CD4^+^ T cells, but not CD8^+^ T cells, required CD40-CD40L costimulation. Most importantly, antifungal CD8^+^ T cells failed to become activated and mediate resistance in CD40^−/−^ and CD40L^−/−^ mice when CD4^+^ T cells were present, indicating that the presence of CD4^+^ T cells impeded priming of CD8^+^ T cells (24). In this study, CD8^+^ T cells acquired and mediated vaccine immunity in the presence of CD4^+^ T cells, but depletion of CD4^+^ T cells during the fungal challenge further augmented the number and function (cytokine production) of CD8^+^ T cells. Thus, the prominent role of CD8^+^ T cells over CD4^+^ T cells in mediating Advax3-induced vaccine immunity was unanticipated and merits further investigation. Our results raise the possibility that the Advax3 adjuvant was key to this observation, due to its ability to strongly prime CD8^+^ T cells, thereby preventing normal CD4^+^ T-cell suppression. Consistent with our findings in the present study, Advax2 and -3 have both been shown to induce cross presentation to CD8^+^ T cells and induce high levels of *in vivo* cytotoxic T-lymphocyte (CTL) killing of CD8 peptide-labeled target cells (11, 25). Our findings on the apparent ability of Advax3 to directly engage CD8^+^ T cells in mediating antifungal vaccine protection even in the absence of CD4^+^ T cells could have important implications for successful immunization of patients with compromised CD4^+^ T-cell immunity who are vulnerable to fungal and other microbial infections, including bacterial pneumonia, in which an unexpected role for CD8^+^ T cells (26) and a dispensable role for CD4^+^ T cells (27) have been described.

## MATERIALS AND METHODS

### Fungi.

Wild-type, virulent B. dermatitidis ATCC strain 26199 was used for this study and grown as yeast on Middlebrook 7H10 agar with oleic acid-albumin complex (Sigma) at 39°C.

### Mouse strains.

Inbred wild-type C57BL/6 mice obtained from Jackson Laboratories were bred at our facility. Male and female mice were 7 to 8 weeks old at the time of these experiments. Mice were housed and cared for as per guidelines of the University of Wisconsin Animal Care Committee, who approved all aspects of this work.

### Vaccination and infection.

For Advax comparisons, 10 μl of Advax adjuvant (Advax1 to -9) was combined individually with 10 μg of soluble Bl-Eng2 protein and diluted to 200 μl with phosphate-buffered saline (PBS). The vaccine was delivered in this 200-μl dose by subcutaneous injection. Mice received one boost 2 weeks following the initial vaccination.

In adjuvant comparison experiments, Bl-Eng2 was formulated in complete and incomplete Freund’s adjuvant (CFA and IFA, respectively), 10% Adjuplex, and Advax3. Fifty microliters of CFA was combined with 10 μg of soluble Bl-Eng2 protein and diluted to 200 μl with PBS. CFA vaccines were then sonicated and injected subcutaneously in 200-μl doses. Adjuplex vaccines were also delivered in 200-μl doses by adding 10 μg of soluble Bl-Eng2 protein to 20 μl of Adjuplex adjuvant and diluting to 200 μl with PBS. Advax3 was formulated as described above. Following the initial vaccination, mice received two subsequent boosts with IFA, 10% Adjuplex, and Advax3, spaced 2 weeks apart.

Vaccinated animals were infected with B. dermatitidis yeast 2 weeks after the last boost. The mice received 2 × 10^4^ yeast by aspiration as described previously (19). Mice were euthanized for cellular and CFU analysis either at day 4 postinfection or when they first appeared moribund.

### T-cell stimulation and flow cytometry.

The lungs were dissociated in Miltenyi magnetically activated cell sorting (MACS) tubes and digested with collagenase D (1 mg/ml) or collagenase B (2 mg/ml, for influenza) and DNase (1 μg/ml) for 25 min at 37°C. The digested lungs were resuspended in 5 ml of 40% Percoll (GE Healthcare, catalog no. 17-0891-01); 3 ml of 66% Percoll was underlaid. Samples were spun for 20 min at 2,000 rpm at room temperature. The lymphocyte layer was collected and resuspended in complete RPMI (10% fetal bovine serum [FBS] and 100 μg/ml penicillin-streptomycin). For the influenza vaccine model, the Percoll separation was not performed. Instead after digestion of the lungs, the red blood cells were lysed with ACK buffer, the samples were filtered in 40-μm-pore cell strainers, and the cells were resuspended in complete RPMI. For *ex vivo* stimulation, lung T cells were incubated at 37°C for 5 h with 5 μM Bl-Eng2 peptide and 1 μg antimouse CD28 (BD, catalog no. 553294). After 1 h, GolgiStop (BD, catalog no. 554724) was added to each well. All fluorescence-activated cell sorter (FACS) samples were stained with LIVE/DEAD Fixable Near-IR Dead Cell stain (Invitrogen) and Fc Block (BD) for 10 min at room temperature. T cells were stained with Bl-Eng2 tetramer for 1 h at room temperature. Then, the cells were stained for surface antigens and intracellular cytokines. All panels included a dump channel (B220, CD11b, CD11c, and NK1.1). Fifty microliters of AccuCheck counting beads (Invitrogen, catalog no. PCB100) was added to the samples to determine the absolute cell count. Samples were acquired on a LSR Fortessa at the University of Wisconsin Carbone Cancer Center Flow Lab.

### Surface panel cocktail for Bl-Eng2 peptide experiments.

The following antibodies were used in a surface panel cocktail for the Bl-Eng2 peptide experiments: anti-CD45–Alexa Fluor 488 (clone 30-F11; Biolegend, catalog no. 103122), anti-major histocompatibility complex (MHC) class II tetramer–phycoerythrin (PE), anti-CD4–BUV395 (clone GK1.1; BD, catalog no. 563790), anti-CD8–peridinin chlorophyll protein (PerCP)-Cy5.5 (clone 53-6.7; Biolegend, catalog no. 100734), anti-CD44–BV650 (clone IM7; Biolegend, catalog no. 1033049), anti-CD11b–allophycocyanin (APC) (clone M1/70; Biolegend, catalog no. 101212), anti-CD11c–APC (clone N418; Biolegend, catalog no. 117310), anti-NK1.1–APC (clone PK136; Biolegend, catalog no. 108710), and anti-B220–APC (clone RA3-62B; Biolegend, catalog no. 103212).

### Lung histology.

Random sections of lungs were harvested and fixed in 10% buffered paraformaldehyde for at least 48 h. Fixed samples were dehydrated with a graded ethanol series, embedded in paraffin, and sectioned at a thickness of 5 μm with a rotary microtome. Sections were stained using the Mayer’s hematoxylin and eosin (H&E) method. Histological sections were assessed for lung inflammation under light microscopy in a blind fashion.

### *In vivo* T-cell depletion and cytokine neutralization.

Vaccinated mice were depleted of T-cell subsets by intravenous (i.v.) injection of 100 μg anti-CD4 (clone GK1.5) and/or anti-CD8 (clone 53-6.7) or rat IgG as a negative control 2 h before challenge and weekly thereafter. Cytokines were neutralized by i.v. injection of 100 μg anti-IFN-γ (clone XMG1.2) and/or anti-IL-17A (clone 17F3) MAbs 2 h before challenge and every other day thereafter. Treg cells were depleted by treating mice with anti-CD25 MAb at days −7 and −4 of infection and anti-CTLA4 MAb at the time of infection and weekly thereafter as previously described (21). All antibodies were purchased from BioXcell, Lebanon, NH, USA.

### Statistics.

All statistics for flow cytometry and CFU were calculated in Prism (GraphPad). An unpaired two-tailed *t* test was used to calculate significance of differences between naive and vaccinated groups in either absolute number or percentage of cells. For comparisons of absolute cell counts, data were log transformed before *t* tests were conducted. CFU data were also log transformed, and an unpaired two-tailed *t* test with Welch’s correction was used to calculate *P* values. Survival outcomes were analyzed in Prism (GraphPad) using the Bonferroni method adjusting for multiple comparisons. A value of *P* ≤ 0.05 was considered statistically significant.

### Ethics statement.

The animal studies performed were governed by protocol M005891 as approved by the IACUCs of the University of Wisconsin—Madison Medical School. Animal studies were compliant with all applicable provisions established by the Animal Welfare Act and the Public Health Services (PHS) Policy on the Humane Care and Use of Laboratory Animals.
